# *Ex situ* co culturing of the sea urchin, *Mespilia globulus* and the coral *Acropora millepora* enhances early post-settlement survivorship

**DOI:** 10.1038/s41598-019-49447-9

**Published:** 2019-09-10

**Authors:** Jamie Craggs, James Guest, Mark Bulling, Michael Sweet

**Affiliations:** 1 Aquatic Research Facility, Environmental Sustainability Research Centre, University of Derby, Derby, DE22 1GB United Kingdom; 2 Horniman Museum and Gardens, Forest Hill, London, SE23 3PQ United Kingdom; 3 School of Natural & Environmental Sciences, Newcastle University, Newcastle upon Tyne, NE17RU United Kingdom

**Keywords:** Environmental impact, Marine biology

## Abstract

Reef restoration efforts, utilising sexual coral propagation need up-scaling to have ecologically meaningful impact. Post-settlement survival bottlenecks, in part due to competitive benthic algae interactions should be addressed, to improve productivity for these initiatives. Sea urchins are keystone grazers in reef ecosystems, yet feeding behaviour of adults causes physical damage and mortality to developing coral spat. To investigate if microherbivory can be utilised for co-culture, we quantitatively assessed how varying densities of juvenile sea urchins *Mespilia globulus* (Linnaeus, 1758), reared alongside the coral *Acropora millepora* (Ehrenberg, 1834) effected survival and growth of coral recruits. Spawning of both species were induced *ex situ*. A comparison of *A. millepora* spat reared in three *M. globulus* densities (low 16.67 m^−2^, medium 37.50 m^−2^, high 75.00 m^−2^) and a non-grazed control indicated coral survival is significantly influenced by grazing activity (*p* < 0.001) and was highest in the highest density treatment (39.65 ± 10.88%, mean ± s.d). Urchin grazing also significantly (*p* < 0.001) influenced coral size (compared to non-grazing control), with colonies in the medium and high-densities growing the largest (21.13 ± 1.02 mm & 20.80 ± 0.82, mean ± s.e.m). Increased urchin density did however have a negative influence on urchin growth, a result of limited food availability.

## Introduction

Anthropogenic driven climate change is causing significant loss of associated biodiversity in coral reef habitats^1–3^, resulting in the global decline of these ecosystems^4^. This has led some researchers to suggest that human intervention, through active restoration, will be increasingly important^5,6^.

Transplantation of scleractinian corals on damaged reefs has been used widely as a tool for restoration for nearly three decades^7^. Transplantation of corals that have been reared from asexually derived fragments, is considered a relatively low cost restoration technique and can be implemented with little training^8,9^. However, this approach has several limitations. For example, the use of asexual fragments results in limited genetic diversity of restored populations, creating potentially undesirable consequences such as reduced population resilience when subjected to negative environmental stress^10^.

**Figure 1:**
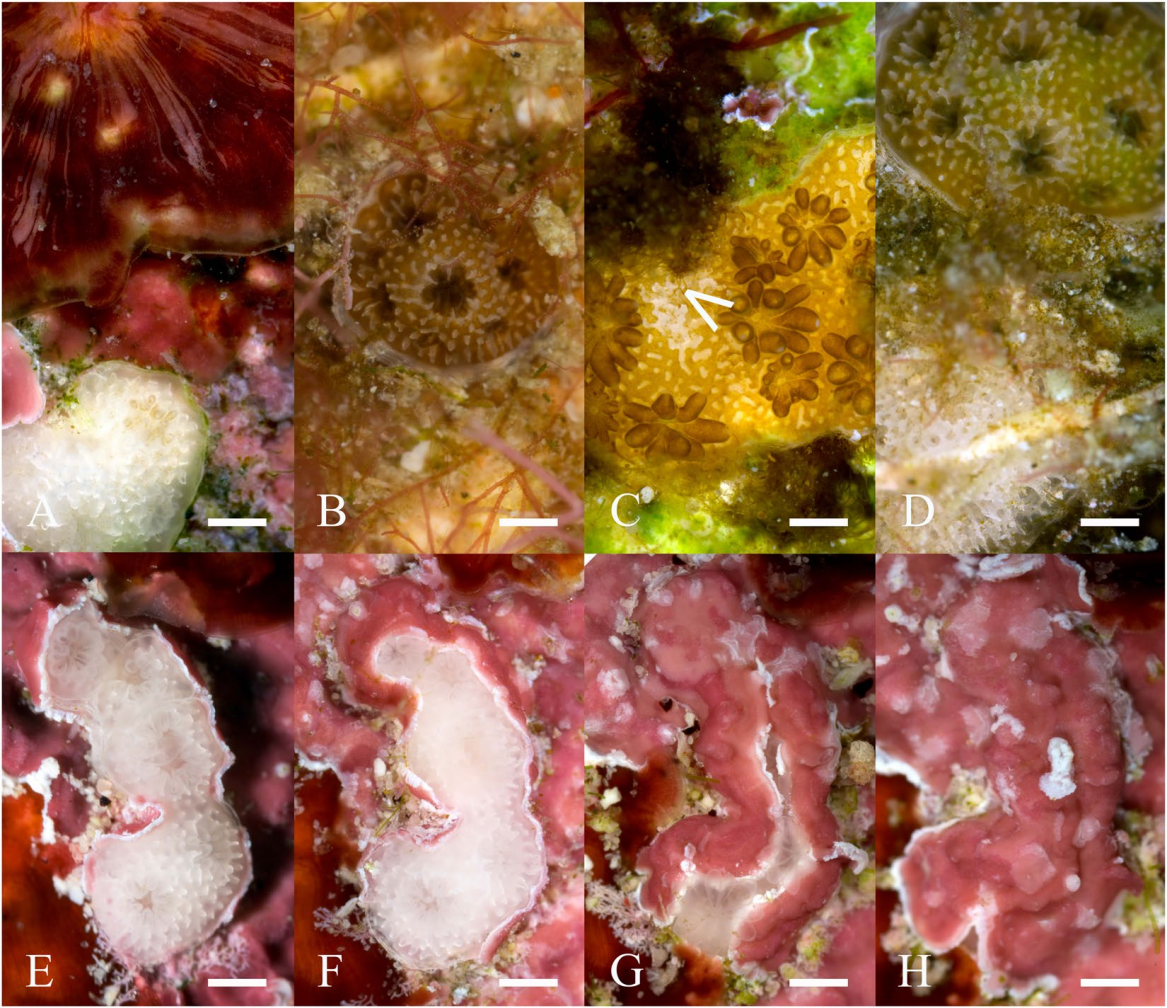
Competitive benthic interactions causing juvenile coral mortality. (**A**) *Peyssonnelia squamaria* rapidly over grows juvenile coral; (**B**) filamentous algae encroaching on *Acropora millepora*; (**C**) cyanobacteria and diatom growth causing onset of tissue loss (<) in *Acropora millepora*; (**D**) sediment accumulation around the peripheral edge of a juvenile *Acropora millepora*; (**E**) Unidentified crustose coralline algae overgrowing *Acropora hyacthinus* primary polyps on 19/04/16; (**F**) 25/04/16; (**G**) 4/05/16; (**H**) 9/05/16. Scale 1 mm.

In contrast, production of sexually produced spat circumvents this issue via the genetic recombination and production of new coral genotypes^5,11^. This has led to the development of techniques using sexually propagated corals for production of transplants, ensuring increased genetic heterogeneity in the transplanted cohort^12–14^. However, this in itself fails to address the larger issues associated with restoration projects which is one of scale, i.e., how do we propagate sufficient numbers of corals to counter the loss occurring through anthropogenic degradation? To date *ex situ* sexually reproduced coral spat have only be transplanted onto small areas (i.e., 10’s m^2^) and costs of undertaking these approaches remains high. Indeed, estimates range from US$4.4 to US$60 per coral depending on the method used and the scale of production^12,15,16^. There is therefore an urgent need to explore options of up-scaling methods to meet demand. New techniques have recently been developed that enable large scale production of sexually produced coral spat for just such scenarios^17^. This could then be linked into current more elaborate attempts around reef restoration including practices such as assisted gene flow^18,19^, hybridisation^20^ and the use of coral probiotics^21,22^. When coupled with the advancements in settlement substrates^23^ – upscaling seems to be within reach. However, the cost of such procedures is still argued to be a major hurdle necessary to overcome before true feasibility of reef restoration on a global scale can be reached. For example, whilst the production of large amounts of planula via *in-vitro* fertilisation is now possible, upscaling efforts are compounded by the fact that reef building corals undergo a survival bottleneck following fertilisation and embryogenesis, with high mortality rates being recorded during the early life history stages when the newly settled spat are small and vulnerable^12,24–27^. Mortality can occur for a broad range of reasons, from chronic stressors such as benthic competition and predation, to more acute impacts associated with bleaching and disease^28^. Newly settled coral appear to have a limited ability to deal with competitive benthic interactions^29^. For example, overgrowth by algae^30,31^ (Fig. 1A–C), damage via sedimentation^32^ (Fig. 1D) and encrusting invertebrates^33^, all regularly impact survival at this early life stage.

The important contribution of heterotrophic feeding to spat survivorship has recently been highlighted (especially during early ontogeny), and it has been argued that reducing mortality rates in these early post settlement stages is key here as well^34^. Therefore, the concept of ‘co-culturing’ could be utilised to address these issues^14,35^. Indeed, Villanueva *et al*.^36^ increased coral spat survivorship when juveniles were co-cultured with the herbivorous gastropod *Trochus niloticus*. Turf algae growth was lowered with increased grazing density and resulted in a 13% increase in survivorship when compared to ungrazed surfaces, five weeks post settlement. However, while *T. niloticus* are effective grazers of soft filamentous algae, they are unable to control macroalgae, ruffed algae or crustose coralline algae (CCA)^37,38^. The latter issue could be particularly important for post settlement survivorship in coral recruits. Whilst CCA (and the bacteria associated with them) provide the settlement cue for coral planula larvae to metamorphosis from their pelagic to benthic stage^39–41^ many species exhibit varying degrees of anti-settlement strategies such as epithellial shedding, overgrowth (Fig. 1E–H), and potential chemical deterrents which have a direct impact on coral post settlement survival^33^.

Coral recruitment has been shown to increase on reefs where urchin grazing is high^42^ and where CCA growth is heavily grazed^43^. Herbivore size appears to play a key role in coral recruit survivorship^44–46^, yet there is no experimental evidence to assess the influence on growth and survivorship of microherbivory in controlled conditions. In the present paper we successfully co-cultured the sea urchin *Mespilia globulus* and the hard coral *Acropora millepora*. We then tested the effect of different densities of urchin on early survivorship of coral spat.

## Results

### Influence of grazing density on coral survivorship

**Figure 2:**
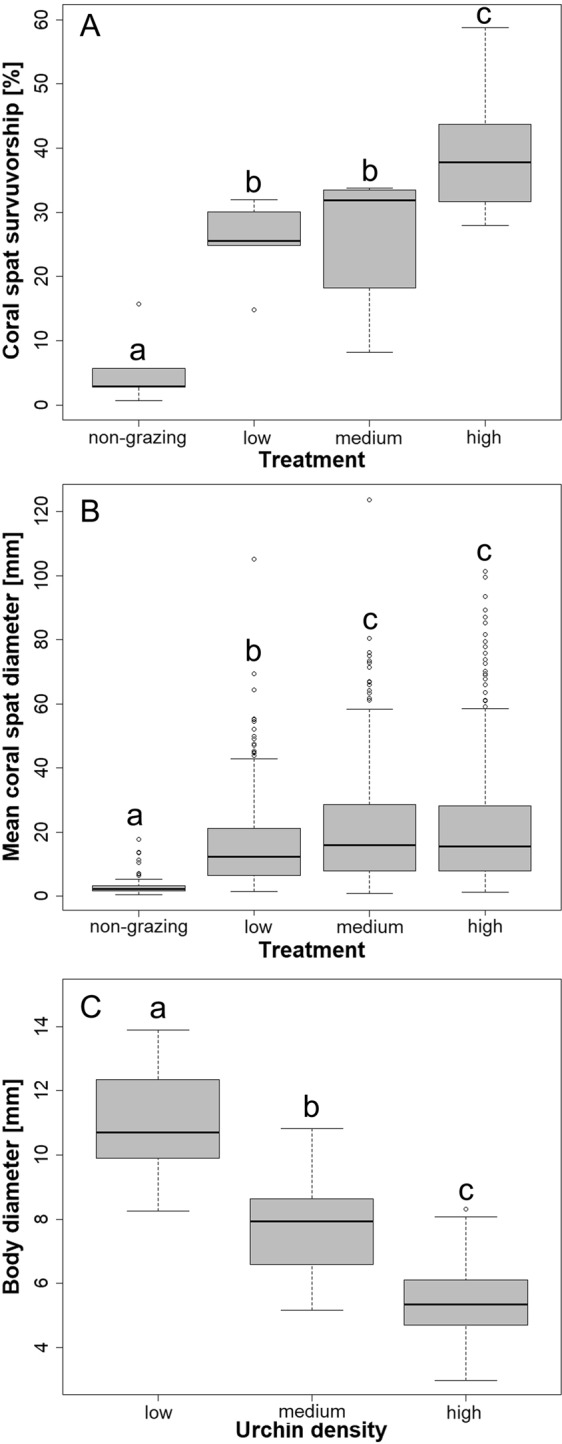
(**A**) *Acropora millepora* percentage (mean ± s.e.m) spat survivorship at 180 days post settlement; (**B**) *A millepora* spat diameter (mean ± s.e.m) at 180 days; (**C**) *Mespilia globulus* body diameter (mean ± s.e.m) at 180 days. [Non-grazing control, low grazing density (four urchins = 16.67 m^−2^), medium grazing density (nine urchins = 37.50 m^−2^) and high grazing density (18 urchin = 75.00 m^−2^)]. The boxplots show the median (black line), the first and third quartiles (grey shaded box), and the lower and upper extremes, circles represent suspected outlying values. Different letters indicate significant differences between means (Linear regression, p < 0.05).

**Figure 3:**
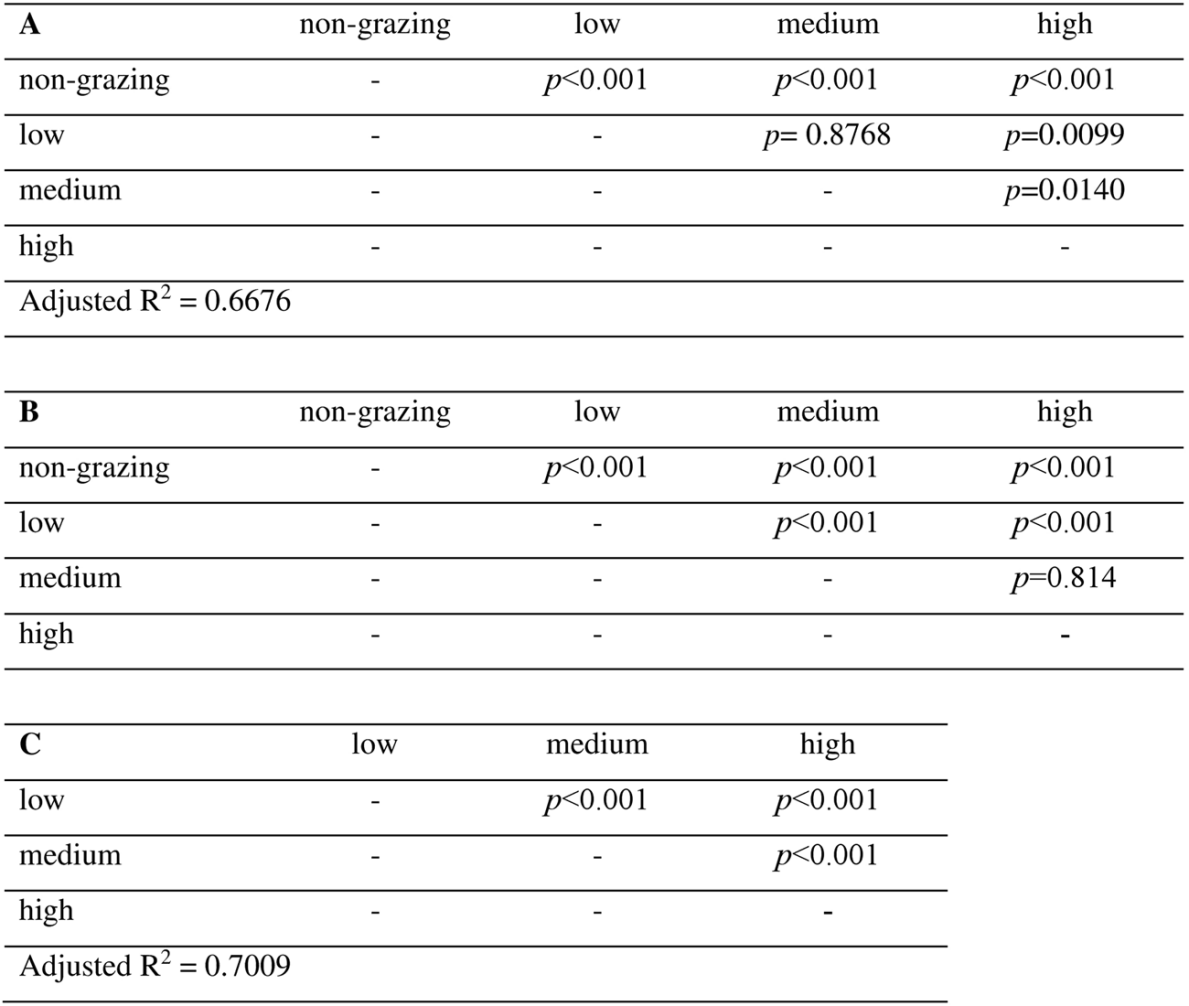
(**A**) Effects of *Mespilia globulus* grazing density on *Acropora millepora* spat survivorship at 180 days from linear regression models (LM); (**B**) *A. millepora* colony diameter from linear regression with GLS extension; (**C**) *M. globulus* basal diameter from LM at 180 days based. [Non-grazing control, low grazing density (four urchins = 16.67 m^−2^), medium grazing density (nine urchins = 37.50 m^−2^) and high grazing density (18 urchin = 75.00 m^−2^)].

Juvenile urchin grazing had a significant effect on coral survival in all grazed treatments (low, medium and high) compared to the non-grazing control at day 180 (*p* < 0.001, Fig. 2A, Fig. 3A). Spat survival was greatest in the highest grazing density (39.65 ± 10.88%, mean ± s.d) and lowest in the non-grazed control (5.09 ± 5%) (Fig. 2A). Survivorship at high density was significantly greater than low and medium density treatments (*p* = 0.0099 and *p* = 0.0140 respectfully, Fig. 3A, Fig. 2A). No significant difference was observed between corals in the lowest grazing density and medium grazing density (*p* = 0.877, adjusted R^2^ = 0.67).

**Figure 4:**
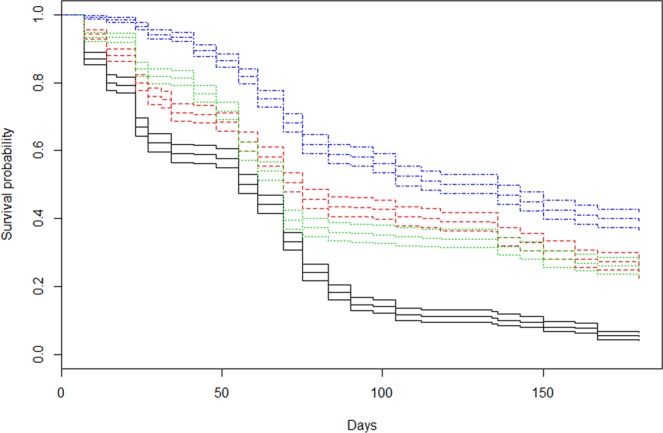
Kaplan-Meier survival curves of newly settled *Acropora millepora* recruits exposed to differing levels of grazing pressures [non-grazing control (black solid line), low grazing density four juvenile *Mespilia globulus* (16.67 m^−2^) (green dotted line), medium grazing density nine juvenile *M. globulus* (37.50 m^−2^) (red dashed line), high grazing density 18 juvenile *M. globulus* (75.00 m^−2^) (blue dash dot line)] and grown over 180 days (mean ± s.e.m).

**Figure 5:**
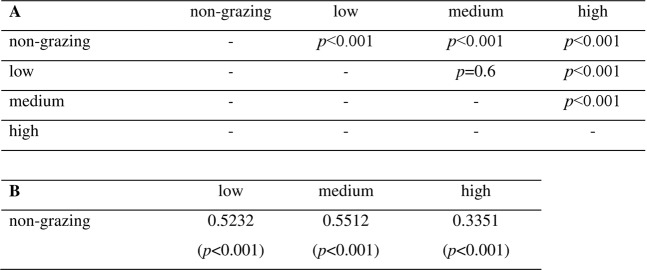
Kaplan-Meier survival showing effects of *Mespilia globulus* grazing density on *Acropora millepora* spat survivorship over 180 days. (**A**) Pairwise log-rank tests differences between treatment survival curves; (**B**) Result of the cox proportional hazard models illustrating exponent coefficient and significance of proportional risks of mortality between individuals across treatments. [Non-grazing control, low grazing density (four urchins = 16.67 m^−2^), medium grazing density (nine urchins = 37.50 m^−2^) and high grazing density (18 urchin = 75.00 m^−2^)].

Pairwise log-rank tests for treatment differences between the Kaplan-Meier coral survival curves (Fig. 4) showed a similar significantly higher survival in all grazed treatments compared to the non-grazed control (*p* < 0.001, Fig. 5A). Coral survival was significantly greater throughout the experiment in the high grazing density compared to both medium and low densities (*p* < 0.001 for both high vs low and high vs medium, Fig. 5A). Again similar to the Linear regression models (LM), no significant difference occurred in the Kaplan-Meier coral survival curves between corals in the low density and medium density (*p* = 0.6, Fig. 5A).

Exponent coefficients from the Cox proportional hazards test confirmed that the probability of coral mortality in low, medium and high grazing density treatments were all lower compared to the non-grazed control (0.523, 0.551 & 0.335 respectfully, Fig. 5B). This highlights that in the highest density treatment, any given coral recruit is approximately a third (0.335) less likely to die than any recruit in the control, non-grazed treatment.

### Influence of grazing density of coral size

**Figure 6:**
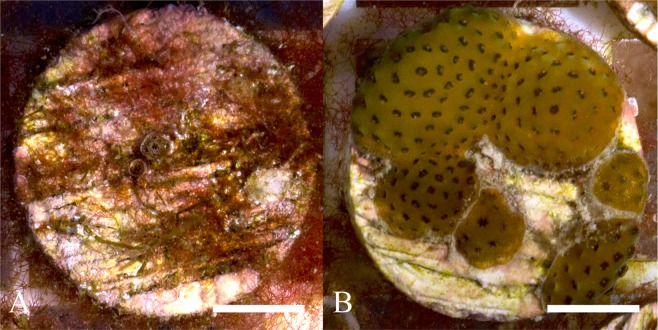
*Acropora millepora* colonies at 180 days post settlement showing coral size comparison between (**A**) non-grazing control and (**B**) high urchin density (75.00 m^−2^). Scale = 5 mm.

Residuals for the model of coral diameter showed heterogeneity of variance, and therefore a generalized least square (GLS) extension was applied (See Supplementary Table S1 for raw data size frequency distributions). Colony size of corals surviving at 180 days was significantly affected by presence or absence of grazing urchins (Fig. 2B, Fig. 6). Those growing in the presence of urchins all attained a significantly larger size compared to the non-grazed control (*p* < 0.001, Fig. 3B). Colony diameter was also found to be largest in the medium and high grazing treatments and were significantly greater than in the low grazing treatment (*p* < 0.001 & *p* < 0.001 respectfully, Fig. 3B).

### Influence of grazing density on urchin growth

Mean body diameter was largest for urchins in the low grazing density treatment (11.01 ± 0.26 mm, mean ± s.e.m), followed by medium grazing density (7.69 ± 0.17 mm). Urchins in the high density treatment grew the least during the 180 day experiment (5.37 ± 0.12 mm) (Fig. 2C). Urchin density influenced basal diameter highlighted by significant differences between all treatments (low vs medium *p* < 0.001, low vs. high *p* < 0.001, and medium vs. high *p* < 0.001, adjusted R^2^ = 0.70, Fig. 2C, Fig. 3C).

## Discussion

Upscaling restoration efforts must focus on enhancing productivity, improved coral resilience and increased yield^19^. Increasing coral survivorship during early propagation is a critical first step as this should dramatically reduce the ‘cost’ of producing a coral that can be transplanted back onto the reef. Here we demonstrate that the sea urchin *Mespilia globulus* can be induced to spawn on demand using rapid temperature shock and, with appropriate planning, reared and grown (*ex situ*) for utilisation as an effective grazing species with coral spat. We show that during co-culturing, urchin density plays an important role in modulating coral survivorship and growth rates. In addition, we show that both species can be reared using a low ‘tech’ transferable methodology with a high level of success.

Comparable to a similar field based study with the gastropod mollusc *Trochus niloticus*^36^, we illustrate increased urchin density resulted in significantly higher coral survivorship over the 180 day period, probably due to the reduced benthic competition from algal species. Although, Villanueva *et al*. (2013) also highlighted improved coral survivorship at higher grazing densities (eight *T. niloticus* m^−2^), their study only spanned five weeks (35 days). Furthermore, survivorship was higher in our current study (39.65 ± 10.88%, mean ± s.d) than that found with *T. niloticus* (18.3% ± 6.7%, mean ± s.d), suggesting urchins are possibly a more effective grazing invertebrate when the goal is increasing coral survivorship.

Increasing urchin density not only enhanced coral survivorship but also resulted in increased coral growth rates. This is also likely accredited to the microherbivory reducing competition with benthic algae and allowing for unimpeded coral growth, a result previously unreported. As larger corals have a greater chance of survivorship (once out planted on the reef^12^), increasing growth rates allows for outplanting sooner and furthers the potential to reduce restoration costs.

Unsurprisingly, whilst coral survival and size were positively affected by increasing urchin density, this appeared to come at the detriment of the urchin itself. As density increased, urchin growth was retarded, probably as a result of limited food availability^47^. What was surprising is that, despite detailed assessment of the health of the recruits, there was no evidence of physical abrasive damage to the corals, this was expected from the rasping actions of urchin grazing. This further suggests survival was enhanced due to the smaller sizes of the urchins i.e. the co-culturing methodology.

Collectively, this study illustrates that a more holistic approach of multi taxa co-culturing can increase the production of sexually diverse coral spat and that if applied to restoration practises, could facilitate up-scaling efforts. The period over which a coral remains in a nursey (prior to transplantation) influences growth, survival and ultimately cost per unit of the transplant, with longer nursery rearing periods leading to lower overall costs per coral^12^. Time invested ‘in nursery’ care therefore plays an important role in the cost per unit and methods to increase production must be an important focus in reducing overall costs of restoration efforts.

It should be noted, that the practice of co-culturing organisms (spanning different trophic levels as undertaken in this study) has been undertaken before. For example in bioremediation of coastal aquaculture initiatives^48^. Lower trophic organisms such as the red alga *Gracilaria lemaneiformis* utilise the waste products, inorganic nitrogen and phosphorus, from higher trophic target species (such as the food fish *Sebastodes fuscescens*)^49^. Primarily, this practice is undertaken in order to reduce negative environmental impact associated with eutrophication^49^. Reid *et al*.^50^, however also highlighted that economic value usually increases when an integrated multi-trophic aquaculture approach is utilised. Such multi-trophic approaches are now clearly achievable with regard to coral reef restoration practices. For example, an early study by Pomeroy *et al*.^51^ investigated the financial and social feasibility of aquaculture of a variety of reef organisms, as an alternative to wild collection. Through analysis of socioeconomic dimensions they concluded that (under certain conditions), such an approach could provide alternative livelihoods to local people and prove useful in reducing fishing pressure on the reefs. The methods illustrated in this study should open up an avenue for self-sustained funding if the co-cultured organisms (urchins in this instance), also have an economic value themselves^52^. The coral *Acropora millepora* and sea urchin *M. globulus* – both utilised in this study are regularly imported across the marine ornamental industry – thus increasing *ex situ* culture of these organisms should reduce demand on wild stocks, with the added benefit of supplementing reef restoration practices.

Targeting more economically important species in any such co-culturing venture would further add to the cost reduction benefits. For example, the collector sea urchin *Tripneustes gratilla* is an important food item in many countries across the world such as the Philippines and South Korea^53^. Whilst not the main focus of this paper we have been successful in culturing *T. gratilla*, and therefore suggest this species as an alternative to *M. globulus* when co-culturing corals in future projects. See Supplementary Fig. S1 for the developmental stages of *T. gratilla*, which may prove a useful guide for assessing health state when implementing this practice. Once juvenile *T. gratilla* have surpassed their ‘useful size’ for improved coral spat survivorship they could be grown on for the secondary process of roe production, thereby increasing financial benefit of reef restoration and creating a circular economy, effectively self-sustaining reef restoration with funds from aquaculture practices^54^.

Another possibility would be the application of co-culturing for multi-taxa restoration. In the Caribbean for example, two coral species *Acropora palmata* and *Acropora cervicornis*, both listed as Critically Endangered by the IUCN Red List of Threatened Species, along with the key stone herbivore, *Diadema antillarum*, that suffered massive declines in populations during the early 1980s^55–57^ would make prime candidates. To date, only limited success has been had in restoration efforts for these species but *ex situ* rearing has been shown to be possible for both the corals and the urchins separately^58–62^. Combining culturing efforts may therefore increase productivity and increase restored ecosystem functionality and should be a focus for future work.

## Materials and Methods

### Co-culturing urchins and corals

#### Urchin spawning and development

**Figure 7:**
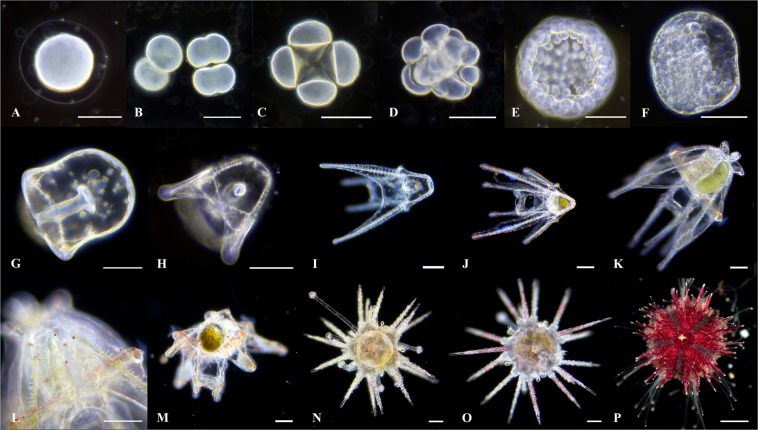
*Mespilia globulus* planktonic development. (**A**) fertilisation membrane surrounding the oocyte 20 mins following oocyte sperm mixing; (**B**) 1^st^ cleavage showing two cell blastomere and zygote undergoing early instigation of second cleavage (0.5 hr post fertilisation (hpf); (**C)** four cell blastomere (1-2 hpf); (**D**) 16 cell blastomere (1–2 hpf); (**E**) blastula (3 hpf); (**F**) cilia have formed and blastula now actively swimming (18 hpf); (**G**) prismatic stage (20 hpf); (**H**) two arm echinopluteus stage (25 hpf); (**I**) four arm echinopluteus stage with ingested *Isochrysis* cells seen in the stomach (3 days post fertilisation (dpf); (**J**) six arm echinopluteus (11 dpf); (**K,L**) eight arm echinopluteus 16 & 22 dpf; (**M**) rudiment formation (22 dpf); (**N**–**P**) 1, 2 and 49 days post settlement. Scale A-O = 100 µm, P = 1 mm.

Ten adult *Mespilia globulus* (19.23 ± 2.03 mm, mean diameter ± s.d) were housed in aquaria at 27.24 ± 0.83 °C, mean ± s.d and salinity of 34.7 ± 1.22ppt, mean ± s.d, for three months and fed daily on a diet of dried algae *Porphyra umbilicalis* and *P. yezoensis* (Julian Sprung’s SeaVeggies®) and live *Caulerpa prolifera* and *C. brachypus*. Spawning was induced using a rapid temperature change three months prior to the planned *Acropora millepora* spawn (see methods below) ensuring adequate time for urchin development prior to commencing the experiment. All adults were transferred from their holding aquarium to a 20 litre aquarium filled with newly mixed sea water (NMSW). This was prepared with reverse osmosis water mixed to 34.0ppt with solar evaporated sea salt (H2Ocean Pro D&D The Aquarium Solution), and heated to 31.5 ± 0.5 °C with 300 watt aquarium heater (Visitherm). Two male urchins released sperm 8–12 mins after heat treatment commenced and one female released eggs 20 mins later. Gametes were periodically agitated by gently stirring for 45 mins to allow fertilisation to occur (Fig. 7A). Ten 1 ml sample counts indicated a total of 270,000 oocytes were released. Fertilised zygotes were separated into three 16 litre conical culture vessels at an average density of 5.67 larvae ml^−1^. Developing embryos are negatively buoyant but delicate in nature, water agitation and aeration was provided with an open ended airline providing a bubble rate of 5 s^−1^. This enabled continuous suspension whilst avoiding physical damage. Embryos remained in these cones for the first 72 hours during which time they received three 50% water exchanges of NMSW by siphoning culture vessel water through a 53 µm mesh ensuring the larvae were left in place.

Embryogenesis was completed three days post fertilisation (Fig. 7B–G) and the prismatic larvae (Fig. 7H) began to feed on microalgae. To support development of four armed to eight armed echinopluteus (Fig. 7I–K) and rudiment development (Fig. 7L,M), the algae *Isochrysis* aff. *galbana* Tahitian strain (Haptophyta), was added, as this has been shown to slow larval development time but increase survival rates compared with other algal food sources^63^. Larvae were fed at a concentration of 50 cells ml^−1^ at a stocking density of one larvae ml^−1^. *Isochrysis* cultures were grown in three, five litre glass demijohns using Guillard F/2 formulation culture medium (Micro Algae Grow, Florida Aqua Farms). The darkest culture per day was selected for harvesting and algae cell density were determined by ocular microscopy using a hemacytometer in order to calculate daily larval feed amounts. Following harvesting, cultures were topped up with NMSW at salinity 29–30 ppt and 10 drops per litre of F2 formula were added. Cultures were sieved through a 25 µm mesh (every 3 days) in order to remove particulates and the demijohns were cleaned with citric acid. Following cleaning they were rinsed with reverse osmosis water and the algae replaced. Aeration was supplied via a 4 mm ridged pipe and cultures lit with two 24 watt T5 tubes (Plant Pro & Marine white, Arcadia) on a 12:12 light cycle. Larval densities were determined every 3–4 days to monitor population survivorship.

On day 10 (post fertilisation), the larvae were transferred from the conical culture vessel into three, six litre kreisel bowls. These bowls had six, six cm diameter wholes cut into the side (below the water surface), which were covered with a 60 µm filter mesh (Supplementary Fig. S2). The bowls sat in a water bath connected to the same system used for the broodstock holding and an open ended airline provided gentle water circulation. The meshed holes in the side of the bowls enabled water parameters inside to match the system via diffusion. Once in these bowls the larvae were fed 200 ml of *Isochrysis* twice daily. LED lighting (XHO 50/50, Reef Brite) above the bowls enabled cyanobacteria and diatoms to grow on the internal walls of the bowls which provided food for the juvenile urchins post settlement. Metamorphosis and subsequent settlement occurred from 21 days post fertilisation (Fig. 7N–P). Mean diameter of the juvenile urchins (n = 186 urchins) was 1.53 mm (±0.57 s.d) 49 days post settlement (Fig. 7P) and prior to introduction with settled coral spat.

### Acropora spawning and development

**Figure 8:**
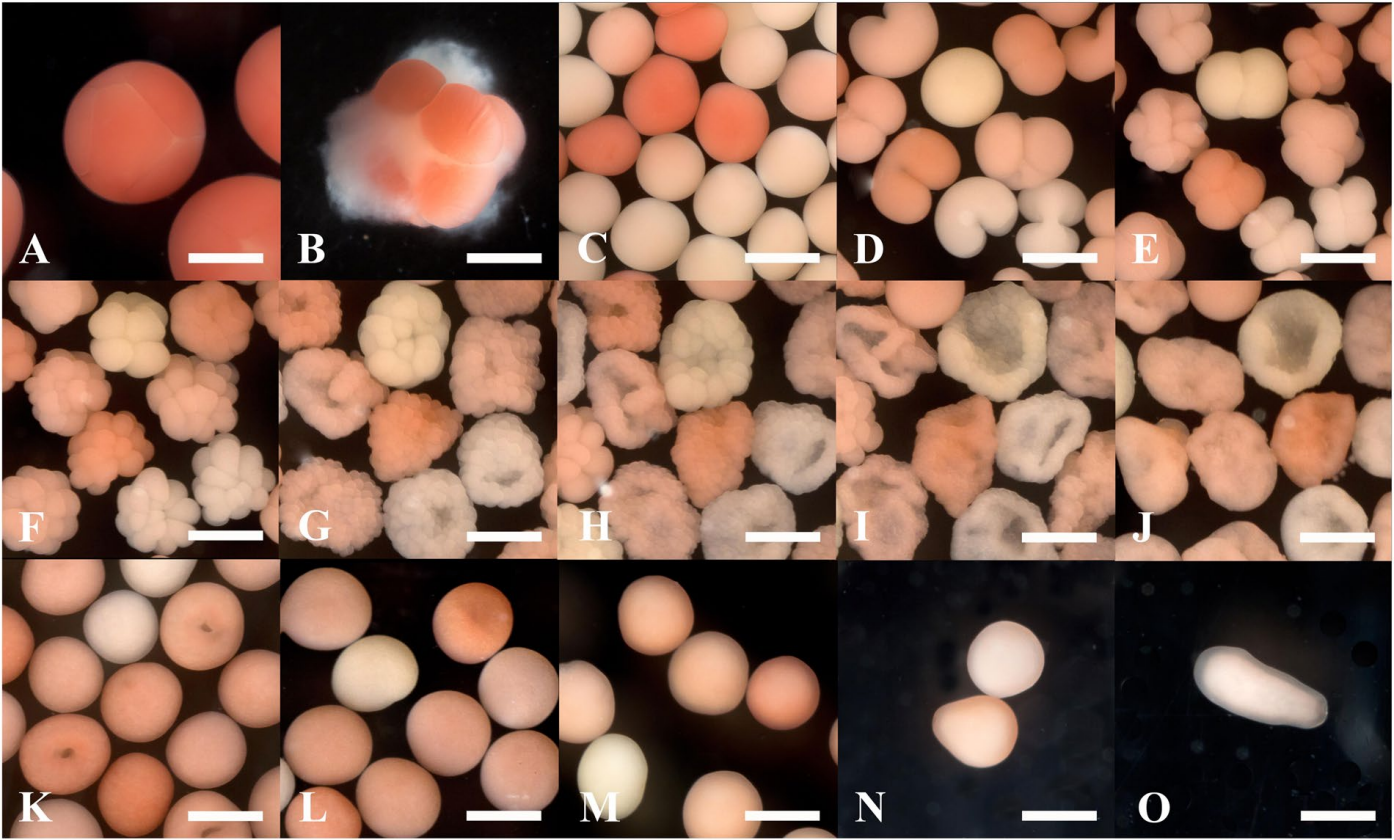
*Acropora millepora* embryogenesis. (**A**) Newly released egg sperm bundles; (**B**) Bundle dissociation occurring, 30–40 mins following release; (**C**) Zygotes following *in-vitro* fertilisation; (**D**) First cell division forming two-blastomere stage, 1–1.5 hr post fertilisation (hpf); (**E**) Four and eight-blastomere stage (2–3 hpf); (**F**) Sixteen-blastomere stage (4 hpf); (**G**) Morula stage (5–6 hpf); (**H,I**) ‘Prawn chip’ stage (6–8 hpf); (**J**) ‘Bowl’ stage (10 hpf); (**K,L**) ‘Round’ stage (18–21 hpf); (**M,N**) ‘Tear drop’ stage (67–70 hpf); (**O**) Planula larval stage (75 hpf). Scale = 500 µm.

*Acropora millepora* colonies were conditioned and induced to spawn *ex-situ* following methods described by Craggs *et al*.^64^. Full gametogenic cycles were therefore able to be completed and spawning time planned to within a window of a few days. Artificial lunar cycles were simulated to coincide with natural cycles with full moon occurring on 4^th^ November 2017. Starting from 10^th^ November, gamete collection rings were placed directly above eight of the conditioned broodstock colonies and held in place with clips. 30 mins prior to the predicted spawning time, the broodstock mesocosm was isolated from the filtration sump below by turning off the main drive pump. In addition, internal water circulations pumps were turned off leaving the water within the mesocosm static. This allowed vertical migration of the buoyant egg sperm bundles^13^ and collection within the rings. Five colonies spawned on 14^th^ November 2017 ten nights after the full moon. Following egg sperm bundle release (Fig. 8A), gametes from all colonies were mixed, divided into ten 50 ml falcon tubes and spun on a rotar at 10 rpm (Maplelab Scientific, model RM-3). This aided bundle dissociation (Fig. 8B). Dissociation was complete when all eggs were separated (46 ± 4 mins s.d). Following, this the speed on the rotator was reduced to six rpm and the tubes left for 45 mins to allow fertilisation to take place. The contents of all tubes were then poured into a 500 ml beaker and the sperm gravity siphoned leaving the zygotes floating at the surface (Fig. 8C). Zygotes were rinsed three times with water from the mesocosm. Following fertilisation embryos were divided into five, six litre kreisel bowls (described earlier). The bowls sat in a water bath connected to the mesocosm to equilibrate temperature and salinity (27.2 degrees and 34.5 ppt respectively). The water height in the bath allowed the bowls to sit above the water surface, preventing the embryos from being lost, but the meshed holes below the water surface (Supplementary Fig. S2) allowing water exchange via diffusion.

First cellular divisions occurred within 2–4 hours (Fig. 8D,E). During the first 24 hrs the bowls were left static with no water input (other than the passive diffusion), allowing the embryos to develop past the fragile ‘prawn chip’ stage^65^ (Fig. 6H–J). 24 hrs post fertilisation water from the mesocosm system was added to the bowls via a 12 ml silicon hose at a flow rate of 200 ml min^−1^. This allowed water quality to be maintained but damage minimised for the remaining time of embryo development (Fig. 6K–N).

The inlet was placed below the water surface and angled to generate a slow circular water movement. If the buoyant developing embryos were dragged down into the water column from the surface the inlet speed was reduced.

Embryogenesis was complete when planula larvae (Fig. 8O) were free swimming at 75 hrs post fertilisation and deemed ready to settle. On 17^th^ November 2017 these were harvested from the five kreisel bowls and placed in a bowl filled (to exactly six litres) with system water from the mesocosm. The water was then randomly stirred with a flat 5 cm × 5 cm piece of UPVC sheet and 10 × 1 ml aliquots were subsequently used to calculate total larval density, which equated to 46,200 larvae. These were introduced to a settlement tank (80 × 40 × 20 cm). This tank contained an egg crate sheet, housing 494 coral settlement plugs (Ocean Wonders, Ceramic Coral Frag Plugs). The plugs had been preconditioned in the main mesocosm for 2 months (prior to the experiment commencing) in order to allow a biofilm to grow on the surface. Five open ended air lines provided aeration and water circulation. As discussed, crustose coralline algae (CCA) aids settlement^39^. Therefore, a mixture of CCA species (*Hydrolithon* spp, *Sporolithon* sp) were ground in a pestle and mortar with NMSW to make a 50 ml solution. This was then sieved through a 53 µm mesh and added to the settlement tank. In order to maintain suitable water quality during the settlement period the settlement tank received a 50% water change daily by placing a 53 µm plankton collector (Florida Aqua Farms inc) into the tank and gravity siphoning water from the inside. The tank was topped back to the same level with water from the main mesocosm. Settlement was completed within 7 days of introduction.

## Experimental Set Up

### Influence of urchin grazing pressure on coral spat survivorship

Density of newly settled echinoids of 255 m^−2^ have been recorded on the central Great Barrier Reef^66^ and peak densities of adults of 73.6 m^−2^ in Hawaii^67^. Therefore, we used this as a proxy to gauge the natural grazing pressure coral recruits would experience *in situ*. Four grazing pressure treatments were trialled, each with six replicate 10 litre tanks (30 × 20 × 20 cm) (n = 24 in total). Treatments included; non-grazing negative control - consisting of no urchins, low grazing density (4 urchins per tank = 16.67 m^−2^), medium grazing density (9 urchins per tank = 37.50 m^−2^), and high or peak grazing density (18 urchins per tank = 75.00 m^−2^). Following coral planula settlement and initial polyp growth, 18 randomly selected settlement plugs were placed into each of the 24 tanks. Initial numbers of coral primary polyps were recorded and totalled 4826 across all treatments (no grazing control n = 1225, low grazing n = 1184, medium grazing n = 1220 and high grazing n = 1197). The 24 tanks were housed in three trough style tanks connected to a centralised mesocosm that housed the broodstock coral colonies. Positioning of the replicates of each treatment were randomly generated to ensure a balanced experimental design. Each tank had a banjo style outlet with an 800 µm mesh. Inlet water from the centralised aquarium system was fed at a flow rate of 91.37 ± 6.80 L.h^−1^ into each of the replicate tanks.

Oocytes and larvae of *Acropora millepora* do not contain zooxanthellae, exhibiting instead horizontal transmission, and acquire Symbiodiniaceae from the water column, post settlement^68,69^. To facilitate this, a single 2–3 cm fragment from the parental colonies was placed into each tank for a period of 9 weeks (and then removed). Each tank was cleaned every fortnight to remove algae from the sides and outlet mesh. As scleractinian corals require heterotrophic feeding for optimal survivorship and growth^34,70^ each tank was dosed three times weekly with 0.1 ml.L^−1^ of amino acid supplement (AcroPower, Two Little Fishes) and live rotifers, *Brachinous plicatilis* (19.15 ± 3.13 rotifer ml^−1^), which had been pre enriched with live *Isochrysis* aff. *galbana* Tahitian strain. During feeding, water supply to the treatment tanks was turned off for approximately 2 hrs. Aeration during this isolate period continued to ensure rotifers were held in suspension to aid prey capture.

### Influence of grazing density of coral percentage survivorship

To assess the impact of grazing pressure on spat survivorship, settlement plugs from all replicates were imaged weekly (Canon 5D Mark III with 100 mm macro lens) for the 180-day duration of the experiment. Coral polyps for each replicate were counted using ImageJ and percentage survivorship for each treatment calculated based on comparison to the first weeks observation for the corresponding replicate.

### Influence of grazing density of coral size

Images taken on day 180, for the coral survival percentage counts were also used to measure colony sizes and assess the influence that urchin density had on coral growth. Coral surface diameter measurements were taken using ImageJ and the fixed coral settlement plug diameter of 19 mm was used as the scale (Fig. 6).

### Influence of grazing density on urchin growth

To determine the influence that grazing density had on urchin growth, individuals from each replicate were imaged on day 180 and body diameter recorded with ImageJ. Urchins from each replica were isolated in a 500 ml dish with a 10 × 10 mm graph paper under for scale reference and imaged three times during the experimental period. The diameter of each urchin (low grazing density – n = 24, medium grazing density – n = 54, high grazing density – n = 108) were determined using the software ImageJ.

### Statistical analysis

LM were used to assess coral percentage survival, coral and urchin size, with the latter parameter utilised as a function of urchin density (treated as a fixed factor). Assumptions of homogeneity and normality were assessed using residual diagnostics, and Cook’s distances were calculated to identify any overly influential data points following Zuur *et al*.^71^. Residuals in the model for coral diameter showed heterogeneity of variance and therefore a GLS extension was applied^72,73^. The most appropriate variance-covariate structure was determined using a combination of AIC scores and plots of fitted values versus residuals for a full model and using restricted maximum likelihood (REML). Backwards stepwise selection was applied using maximum likelihood, with the final minimum adequate model being assessed using REML.

Kaplan-Meier survival curves were estimated for each treatment. To test for treatment differences between survival curves we conducted pairwise log-rank tests. Finally, differences in proportional risks of mortality between individuals in different treatments were tested using a Cox proportional hazards model.

All analyses were conducted using the statistical programming language R (R Development Core Team, 2015) R version 3.3.0 (2016-05-03). The GLS regression used the nlme package^74^, and the survival analyses used the survival package^75^.

## Conclusion

In conclusion, we show that microherbivory can play an important role in increasing coral survival and growth during early ontogeny. The co-culturing methodology used in this study offers significant potential for future coral conservation efforts as it combines enhancing survivorship of coral for transplantation with opportunities to develop sustainable alternative livelihoods and/or support the conservation of threatened urchin species. Future work is now needed to build towards using these methods at larger scales in order to make significant contributions to coral conservation.

## Supplementary information

Supplementary information

## Data Availability

The datasets generated during and/or analysed during the current study are available from the corresponding author on reasonable request.

## References

[1] Richmond RH (1993). Coral reefs: present problems and future concerns resulting from anthropogenic disturbance. Am. Zool..

[2] Carpenter KE (2008). One-Third of Reef-Building Corals Face Elevated Extinction Risk from Climate Change and Local Impacts. Science (80-.)..

[3] Pandolfi JM (2003). Global trajectories of the long-term decline of coral reef ecosystems. Science (80-.)..

[4] Hughes TP (2017). Coral reefs in the Anthropocene. Nature.

[5] Baums IB (2008). A restoration genetics guide for coral reef conservation. Mol. Ecol..

[6] Hoegh-Guldberg O (2007). Coral Reefs Under Rapid Climate Change and Ocean Acidification. Science (80-.)..

[7] Rinkevich B (2005). Conservation of coral reefs through active restoration measures: Recent approaches and last decade progress. Environ.Sci.Technol.

[8] Cruz DW, Villanueva RD, Baria MVB (2014). Community-based, low-tech method of restoring a lost thicket of Acropora corals. ICES J. Mar. Sci..

[9] Bowden-kerby A (2001). Low-tech coral reef restoration methods modeled after fragmentation process. Bull. Mar. Sci..

[10] Omori M (2011). Degradation and restoration of coral reefs: Experience in Okinawa, Japan. Mar. Biol. Res..

[11] Dubinsky Zvy, Stambler Noga (2011). Coral Reefs: An Ecosystem in Transition.

[12] Guest JR, Baria MV, Gomez ED, Heyward AJ, Edwards AJ (2014). Closing the circle: Is it feasible to rehabilitate reefs with sexually propagated corals?. Coral Reefs.

[13] Edwards, A. *et al*. *Reef Rehabilitation. Managing* (2010).

[14] Omori M (2005). Success of mass culture of Acropora corals from egg to colony in open water. Coral Reefs.

[15] Villanueva RD, Baria MVB, dela Cruz DW (2012). Growth and survivorship of juvenile corals outplanted to degraded reef areas in Bolinao-Anda Reef Complex, Philippines. Mar. Biol. Res..

[16] Cruz DWD, Harrison PL (2017). Enhanced larval supply and recruitment can replenish reef corals on degraded reefs. Sci. Rep..

[17] Pollock FJ (2017). Coral larvae for restoration and research: a large-scale method for rearing Acropora millepora larvae, inducing settlement, and establishing symbiosis. PeerJ.

[18] van Oppen MJH, Oliver JK, Putnam HM, Gates RD (2015). Building coral reef resilience through assisted evolution. Proc. Natl. Acad. Sci..

[19] van Oppen MJH (2017). Shifting paradigms in restoration of the world’s coral reefs. Glob. Chang. Biol..

[20] Chan WY, Peplow LM, Menéndez P, Hoffmann AA, van Oppen MJH (2018). Interspecific Hybridization May Provide Novel Opportunities for Coral Reef Restoration. Front. Mar. Sci..

[21] Peixoto RS (2017). Beneficial microorganisms for corals (BMC): Proposed mechanisms for coral health and resilience. Front. Microbiol..

[22] Sweet M, Ramsey A, Bulling M (2017). Designer reefs and coral probiotics: great concepts but are they good practice?. Biodiversity.

[23] Chamberland VF (2017). New Seeding Approach Reduces Costs and Time to Outplant Sexually Propagated Corals for Reef Restoration. Sci. Rep..

[24] Vermeij MJA, Sandin SA (2008). Density-dependent settlement and mortality structure the earliest life phases of a coral population. Ecology.

[25] Babcock R, Mundy C (1996). Coral recruitment: Consequences of settlement choice for early growth and survivorship in two scleractinians. J. Exp. Mar. Bio. Ecol..

[26] Wilson J, Harrison P (2005). Post-settlement mortality and growth of newly settled reef corals in a subtropical environment. Coral Reefs.

[27] Penin L (2010). Early post-settlement mortality and the structure of coral assemblages. Mar. Ecol. Prog. Ser..

[28] Richmond R, Hunter C (1990). Reproduction and recruitment of corals: comparisons among the Caribbean, the Tropical Pacific, and the Red Sea. Mar. Ecol. Prog. Ser..

[29] Ritson-Williams, R. *et al*. New perspectives on ecological mechanisms affecting coral recruitment on reefs. *Smithson. Contrib. Mar. Sci*. 437–457, 10.5479/si.01960768.38.437 (2009).

[30] Harriott VJ (1983). Reproductive seasonality, settlement, and post-settlement mortality of Pocillopora damicornis (Linnaeus), at Lizard Island, Great Barrier Reef. Coral Reefs.

[31] Kuffner IB (2006). Inhibition of coral recruitment by macroalgae and cyanobacteria. Mar. Ecol. Prog. Ser..

[32] Birrell CL, McCook LJ, Willis BL (2005). Effects of algal turfs and sediment on coral settlement. Mar. Pollut. Bull..

[33] Harrington. (2004). Recognition and Selection of Settlement Substrata Determine Post-Settlement Survival in Corals. Ecology.

[34] Conlan JA, Humphrey CA, Severati A, Francis DS (2017). Influence of different feeding regimes on the survival, growth, and biochemical composition of Acropora coral recruits. PLoS One.

[35] Omori M, Kubo H, Kajiwara K, Matsumoto H, Watanuki A (2006). Rapid recruitment of corals on top shell snail aquaculture structures. Coral Reefs.

[36] Villanueva RD, Baria MVB, dela Cruz DW (2013). Effects of grazing by herbivorous gastropod (Trochus niloticus) on the survivorship of cultured coral spat. Zool. Stud..

[37] Lambrinidis G, Luong-Van Thinh J, Renaud S (1997). The growth of juvenile Trochus niloticus fed on algae. ACIAR Proc..

[38] Ng CSL (2013). Dietary habits of grazers influence their suitability as biological controls of fouling macroalgae in *ex situ* mariculture. Aquac. Res..

[39] Morse ANC (1996). An ancient chemosensory mechanism brings new life to coral reefs. Biol. Bull..

[40] Heyward AP, Negri AJ (1999). Natural inducers for coral larval metamorphosis. Coral Reefs.

[41] Negri AP, Webster NS, Hill RT, Heyward AJ (2001). Metamorphosis of broadcast spawning corals in response to bacteria isolated from crustose algae. Mar. Ecol. Prog. Ser..

[42] Edmunds PJ, Carpenter RC (2001). Recovery of Diadema antillarum reduces macroalgal cover and increases abundance of juvenile corals on a Caribbean reef. Proc. Natl. Acad. Sci..

[43] Bruggemann JH, Kessel AMV, Rooij JMV, Breeman AM (1996). Bioerosion and sediment ingestion by the Caribbean parrotfish Scarus vetula and Sparisoma viride: impications of fish size, feeding mode and habitat use. Mar. Ecol. Prog. Ser..

[44] Mumby PJ (2006). Fishing, trophic cascades, and the process of grazing on coral reefs. Science (80-.)..

[45] Penin L, Michonneau F, Carroll A, Adjeroud M (2011). Effects of predators and grazers exclusion on early post-settlement coral mortality. Hydrobiologia.

[46] Trapon ML, Pratchett MS, Hoey AS, Baird AH (2013). Influence of fish grazing and sedimentation on the early post-settlement survival of the tabular coral Acropora cytherea. Coral Reefs.

[47] Ebert TA (1968). Growth rates of the sea urchin Strongylocentrotus purpuratus related to food availability and spine abrasion. Ecol. Soc. Am..

[48] Pierri C, Fanelli G, Giangrande A (2006). Experimental co-culture of low food-chain organisms, Sabella spallanzanii (Polychaeta, Sabellidae) and Cladophora prolifera (Chlorophyta, Cladophorales), in Porto Cesareo area (Mediterranean Sea). Aquac. Res..

[49] Zhou Y (2006). Bioremediation potential of the macroalga Gracilaria lemaneiformis (Rhodophyta) integrated into fed fish culture in coastal waters of north China. Aquaculture.

[50] Reid GK (2011). Recent developments and challenges for open-water, integrated multi-trophic aquaculture (IMTA) in the Bay of Fundy, Canada. Proc. Can. Freshw. Symp. - Aquac. Canada.

[51] Pomeroy RS, Parks JE, Balboa CM (2006). Farming the reef: Is aquaculture a solution for reducing fishing pressure on coral reefs?. Mar. Policy.

[52] Rhyne Andrew L., Tlusty Michael F., Schofield Pamela J., Kaufman Les, Morris James A., Bruckner Andrew W. (2012). Revealing the Appetite of the Marine Aquarium Fish Trade: The Volume and Biodiversity of Fish Imported into the United States. PLoS ONE.

[53] Toha AHA (2017). Biology of the commercially used sea urchin Tripneustes gratilla (Linnaeus, 1758) (Echinoidea: Echinodermata). Ocean. Life.

[54] Mos, B., Cowden, K. L., Nielsen, S. J. & Dworjanyn, S. A. Do cues matter? Highly inductive settlement cues don’t ensure high post-settlement survival in sea urchin aquaculture. PLoS One 6 (2011). 10.1371/journal.pone.0028054 PMC3230603 22162755

[55] Lessios HA (1984). Mass mortality of Diadema antillarum on the Caribbean coast of Panama. Coral Reefs.

[56] Porter JW, Meier OW (1992). Quantification of loss and change in floridian reef coral populations. Integr. Comp. Biol..

[57] Gardner TA, Côté IM, Gill JA, Grant A, Watkinson AR (2003). Long-term region-wide declines in Caribbean corals. Science (80-.)..

[58] Young CN, Schopmeyer SA, Lirman D (2012). a Review of Reef Restoration and Coral Propagation Using the Threatened Genus Acropora in the Caribbean and Western Atlantic. Bull. Mar. Sci..

[59] Quinn NJ, Kojis BL (2006). Evaluating the potential of natural reproduction and artificial techniques to increase Acropora cervicornis populations at Discovery Bay, Jamaica. Rev. Biol. Trop..

[60] Chamberland, *et al*. *Restoration of critically endangered elkhorn coral Acropora plamata populations using larvae reared from wild caught gametes*. 526–537 (2015).

[61] Vaughan, D. *Interim Report: Pilot-Scale Phase (PSP) of Diadema Aquaculture Research Project POR* 2009-30. 1–7 (2010).

[62] Idrisi N, Capo TR, Serafy JE (2003). Postmetamorphic growth and metabolism of long-spined black sea urchin (Diadema antillarum) reared in the laboratory. Mar. Freshw. Behav. Physiol..

[63] Wolcott R, Messing CG (2005). A comparison of diets and water agitation methods for larval culture of the edible sea urchin, Tripneustes ventricosus (Echinodermata: Echinoidea). Bull. Mar. Sci..

[64] Craggs Jamie, Guest James R., Davis Michelle, Simmons Jeremy, Dashti Ehsan, Sweet Michael (2017). Inducing broadcast coral spawning ex situ: Closed system mesocosm design and husbandry protocol. Ecology and Evolution.

[65] Heyward AJ, Negri AP (2012). Turbulance, cleavage and the naked embryo: A case for coral clones. Science (80-.)..

[66] Keesing JK, Cartwright CM, Hall KC (1993). Measuring settlement intensity of echinoderms on coral reefs. Mar. Biol..

[67] Ogden N, Ogden JC, Abbott IA (1989). Distribution abundance and food of sea urchins on a leeward Hawaiian reef. Bull. Mar. Sci..

[68] Little AF, van Oppen MJH, Willis BL (2004). Flexibility in algal endosymbioses shapes growth in reef corals. Science (80-.)..

[69] Van Oppen MJH, Palstra FP, Piquet AMT, Miller DJ (2001). Patterns of coral-dinoflagellate associations in Acropora: Significance of local availability and physiology of Symbiodinium strains and host-symbiont selectivity. Proc. R. Soc. B Biol. Sci..

[70] Petersen D, Wietheger A, Laterveer M (2008). Influence of different food sources on the initial development of sexual recruits of reefbuilding corals in aquaculture. Aquaculture.

[71] Zuur Alain F., Ieno Elena N., Smith Graham M. (2007). Analysing Ecological Data.

[72] Pinheiro & Bates, D. M. Mixed-Effects Models in S and S-PLUS, 10.1007/b98882 (Springer-Verlag, 2000).

[73] West, B. T., Welch, K. B., Gałecki, A. T. & Gillespie, B. W. Linear mixed models: a practical guide using statistical software. (2007).

[74] Pinheiro J (2018). Linear and non-linear mixed effects models. Package ‘nlme’, version.

[75] Therneau, T. M. A Package for Survival Analysis in S. version 2.38. (Springer, 2015).

