# Supplementary material for: Ex situ co culturing of the sea urchin, Mespilia globulus and the coral Acropora millepora enhances early post-settlement survivorship
Source: Sci Rep. 2019 Sep 10;9:12984. doi: 10.1038/s41598-019-49447-9 (PMC6737180; doi:10.1038/s41598-019-49447-9)
Supplement: Supplementary file 1 — Supplementary information [file 41598_2019_49447_MOESM1_ESM.pdf]

Ex situ co culturing of the sea urchin, *Mespilia globulus* and the coral *Acropora millepora* enhances early post-settlement survivorship.

Craggs, Jamie[1,2], Guest, James[3], Bulling, Mark[1] & Sweet, Michael[1]

[1]Aquatic Research Facility, Environmental Sustainability Research Centre, University of Derby, Derby, DE22 1GB, United Kingdom.

[2]Horniman Museum and Gardens, Forest Hill, London, SE23 3PQ, United Kingdom.

[3]School of Natural & Environmental Sciences, Newcastle University, Newcastle upon Tyne, NE17RU, United Kingdom.

Correspondence to jcraggs@horniman.ac.uk

Supplementary Information

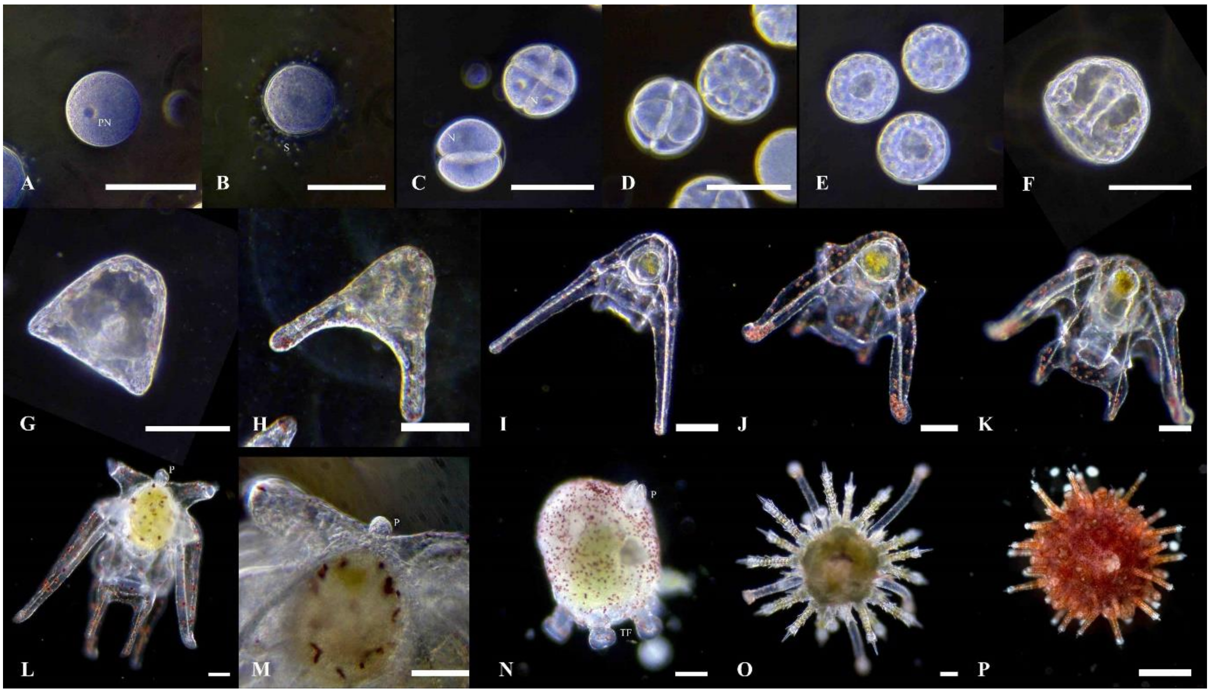

Supplementary Figure S1 – *Tripneustes gratilla* planktonic development. (A) Newly released oocyte showing pronucleus (PN); (B) Spermatozoa surrounding the oocyte 35 mins following oocyte sperm mixing; (C) 1st and 2nd cleavage, two and four cell blastomere with nuclei visible (N) (0.5 – 1.5 hr post fertilisation (hpf); (D) Eight and 16 cell blastomeres (2 hpf); (E) Blastula (4.5 hpf); (F) Cilia have formed, blastula now actively swimming (18 hpf); (G) Prismatic stage (20 hpf); (H) Two arm echinopluteus stage (2 days post fertilisation, dpf); (I) four arm

echinopluteus stage. Ingested *Isochrysis galbana* cells can be seen in the stomach (5 days post fertilisation (dpf); (J) Initialisation of six arm echinopluteus stage (11 dpf); (K) Six arm echinopluteus (12 dpf); (L) Eight arm echinopluteus, with emergence of the first pedicellaria (p) (21 dpf); (M) Enlargement of Figure 5 L showing first pedicellaria (p) (21 dpf); (N) Newly settled "echinoporculus" stage, an intermediate stage between the larvae and juvenile. 1 day post settlement (dps); (O) metamorphosed juvenile (7 dps); (P) juvenile (40 dps). Scale A-O = 100µm, P = 1mm.

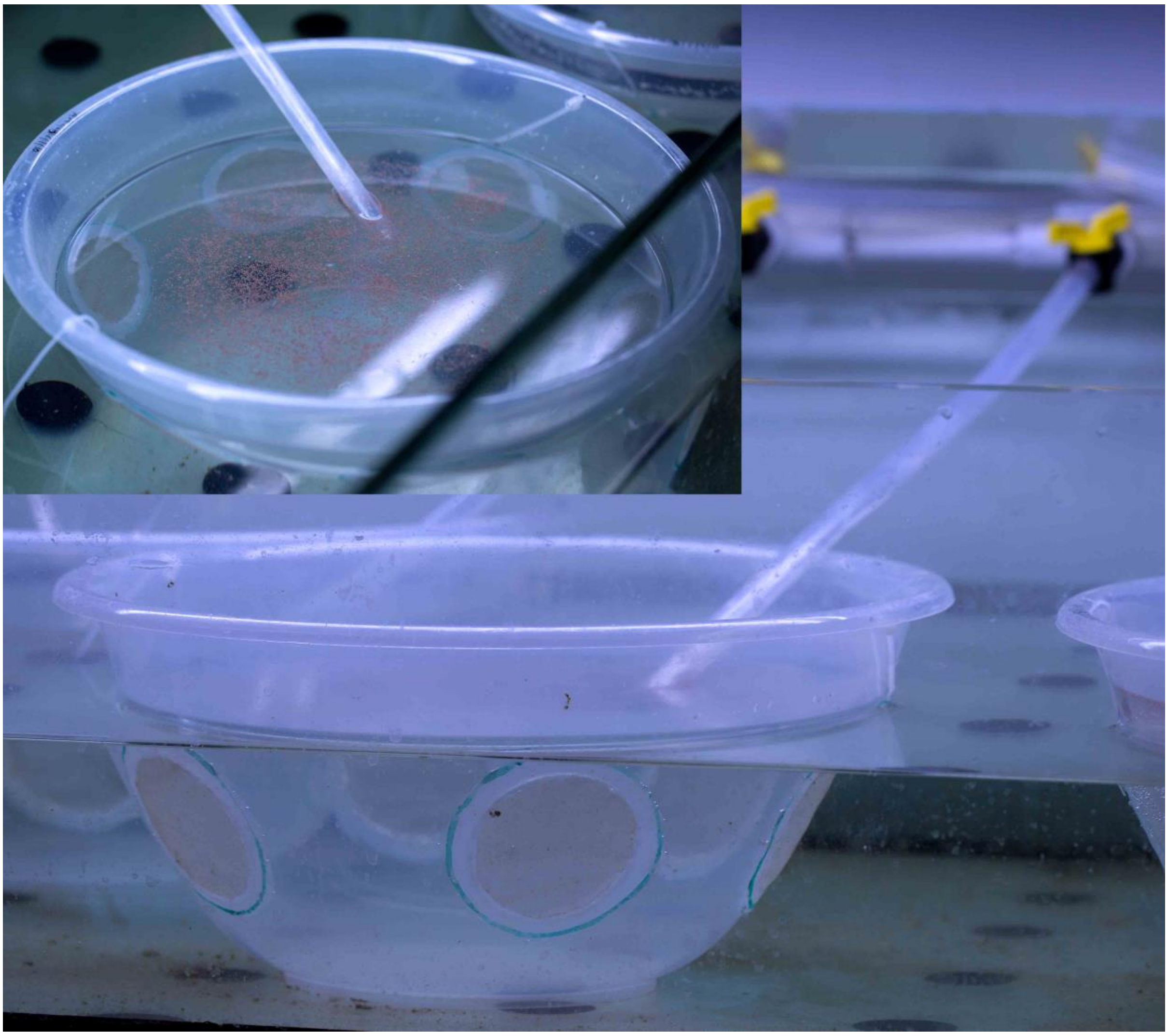

Supplementary Figure S2. Kreisel bowls used to rear both *Mespilia globulus* larvae and *Acropora millepora* embryos. The bowls to sit proud of the water surface preventing the loss of larvae / embryos. 60 µm meshed holes facilitate maintenance of water quality via diffusion.

Supplementary Table S1. Coral size frequency distributions at 180 days in four sea urchin grazing treatments. [non grazing control, low grazing density (4 urchins = 16.67 m^{-2}), medium grazing density (nine urchins = 37.50 m^{-2}), high grazing density (18 urchin = 75.00 m^{-2})].

Coral diameter (mm)	non-grazing	low	medium	high
[0,10]	60	129	95	156
[10,20]	5	82	91	122
[20,30]	0	45	49	83
[30,40]	0	18	28	38
[40,50]	0	14	22	35
[50,60]	0	4	8	9
[60,70]	0	2	7	7
[70,80]	0	0	6	6
[80,90]	0	0	1	4
[90,100]	0	0	0	2
[100,110]	0	1	0	1
[110,120]	0	0	0	0
[120,130]	0	0	1	0
